# Distinguishing very high-risk patients among high-risk gastrointestinal stromal tumor cases: development and validation of a nomogram based on a multicenter population-based retrospective cohort study

**DOI:** 10.1080/07853890.2025.2520896

**Published:** 2025-06-20

**Authors:** Qi Jiang, Yang Fu, Xinhua Zhang, Peng Zhang, Zhidong Gao, Jun Zhang, Xuefeng Zhao, Haibo Qiu, Zilong Zhang, Yingfeng Fu, Jian Liu, Zhenhua Yang, Bo Zhang, Xiaodong Gao, Kaixiong Tao

**Affiliations:** a Department of Gastrointestinal Surgery, Union Hospital, Tongji Medical College, Huazhong University of Science and Technology, Wuhan, China; b Department of Gastrointestinal Surgery, The First Affiliated Hospital of Zhengzhou University, Zhengzhou, China; c Department of Gastrointestinal Surgery, The First Affiliated Hospital, Sun Yat-Sen University, Guangzhou, China; d Department of Gastrointestinal Surgery, Peking University People’s Hospital, Peking University, Beijing, China; e Department of Gastrointestinal Surgery, The First Affiliated Hospital of Chongqing Medical University, Chongqing, China; f The Third Department of General Surgery, The Fourth Affiliated Hospital, Hebei Medical University, Shijiazhuang, China; g Department of Gastric and Pancreatic Surgery, Sun Yat-Sen University Cancer Center/State Key Laboratory of Oncology in South China, Guangzhou, China; h Department of Gastrointestinal Surgery, Affiliated Jingzhou Hospital, Yangtze University, Jingzhou, China; i Department of Gastrointestinal Surgery, Taihe Hospital of Hubei University of Medicine, Shiyan, China; j Department of Gastrointestinal Surgery, The First People’s Hosptical of Jingmen, Jingmen, China; k Department of Gastiointestinal Surgery, Institute of Digestive Disease, China Three Gorges University, Yichang Central People’s Hospital, Yichang, China; l Department of Gastrointestinal Surgery, West China Hospital, Sichuan University, Chengdu, China; m Department of General Surgery, Zhongshan Hospital, Fudan University, Shanghai, China

**Keywords:** Adjuvant therapy, gastrointestinal stromal tumors, gene mutations, nomogram, very high-risk

## Abstract

**Background:**

High-risk gastrointestinal stromal tumors (GISTs) are highly heterogeneous. This study aimed to developa nomogram for predicting recurrence in patients with high-risk GISTand to provide guidance for adjuvant therapy.

**Methods:**

Data were retrospectively collected from 971 patients with high-risk GIST who underwent genetic testingat 13 centers in China. A nomogram was constructed in the training cohort and validated in the validation cohort.

**Rseults:**

The training and validation cohorts included 696 and 275 patients, respectively. The nomogram incorporated blood plateletlevels, total protein, tumor location, tumor size, mitotic count, tumor rupture, Ki-67 index, and gene mutations. The C-index and AUC were 0.758 and 0.781 in the training cohort and 0.841 and 0.755, respectively, in the validation cohort. Calibration and DCA curves confirmed favorable discrimination, calibration accuracy, and clinical benefits. Using the nomogram, patients were categorized into a general high-risk groupand a very high-risk group. Among the general high-risk group, no significant difference in RFS was observed between patients who received adjuvant imatinib for 2.5 years or longer. Conversely, in the very high-risk group, patients receiving adjuvant imatinib for five or more years had markedly improved RFS.

**Conclusions:**

Based on the largest nomogram-related multicenter study in high-risk GIST, the nomogram accurately predicted RFS in patients with high-risk GIST and provided guidance on adjuvant therapy for the first time. For general high-risk patients, 3 years of adjuvant imatinib is adequate, whereas very high-risk patients benefit significantly from more than 5 years of adjuvant imatinib.

## Background

Gastrointestinal stromal tumors (GISTs) are one of the most common gastrointestinal mesenchymal tumors, primarily caused by acquired mutations in the *KIT* or *PDGFRA* genes [1]. The modified National Institutes of Health (NIH) classification system, which categorizes primary GIST into very low, low, intermediate, and high risk of recurrence after complete resection, remains the most commonly used prognostic assessment tool [2]. However, significant heterogeneity exists among GISTs. Even within the high-risk category, patients exhibit significant differences in prognosis.

Recent studies have identified very high-risk patients, characterized by relatively poor prognoses, from the patients with high-risk GIST using a range of clinicopathologic factors [3–6]. Furthermore, efforts to predict recurrence in patients with GIST have leveraged nomograms or AI models [7–10]. Despite these contributions, these studies did not adequately incorporate gene mutations and lacked guidance for adjuvant therapy. This multicenter retrospective study constructed a novel nomogram to accurately predicting recurrence in patients with high-risk GIST and analyzed the adjuvant therapy benefits with the aim of providing a reference for prognostic prediction and treatment for patients with GIST.

## Methods

### Patients

This study retrospectively collected data from patients with GIST who were treated at multiple large centers in China between January 2010 and December 2021. The inclusion criteria were as follows: (1) absence of preoperative treatment, (2) complete tumor resection, and (3) high-risk GIST pathologically confirmed according to the modified NIH classification. The exclusion criteria were: (1) lack of genetic testing or unavailability of genetic testing data, (2) insufficient clinicopathologic data, and (3) incomplete follow-up data. Ultimately, 971 patients with high-risk GIST from 13 centers met the eligibility criteria and were included in this study. Each center was treated as an independent unit and these patients were assigned to the training and validation cohorts in a ratio of approximately 5:2. Finally, the four centers containing relatively larger sample sizes were designated as the training cohort and the remaining nine centers as the validation cohort. The study was approved by the Ethics Committee of Union Hospital, Tongji Medical College, Huazhong University of Science and Technology, which confirmed that informed consent should be waived because this study was a retrospective study and did not compromise patient privacy or cause harm to patients, and was conducted in compliance with the Declaration of Helsinki.

### Data collection

The data collected in this study included the following: (1) demographic data: sex, age, and the age-adjusted Charlson comorbidity index (ACCI) [11]. (2) Preoperative hematological indicators and their calculations: white blood cell count (WBC), red blood cell count (RBC), levels of blood platelet (PLT), total protein (TP), platelet–lymphocyte ratio (PLR), neutrophil–lymphocyte ratio (NLR), systemic immune-inflammation index (SII), platelet–albumin ratio (PAR), prognostic nutritional index (PNI), etc. where PNI = serum albumin (g/L) + 5 × lymphocyte count (10^9^/L). (3) Pathological data: tumor location, tumor size, mitotic count, etc. (4) gene mutation data: based on previous studies [12], gene mutations were categorized into six groups: (1) *KIT* exon 9 mutations, (2) *KIT* exon 11 duplications/indel/substitution mutations and deletions involving only one codon, excluding codons 557 and 558, as well as *KIT* exon 13 mutations, and *KIT* exon 17 mutations. (3) *KIT* exon 11 deletions involving codons 557 and 558. (4) *KIT* exon 11 deletions involving two or more codons, excluding codons 557 and 558. (5) *PDGFRA* mutations. (6) wild type, (7) adjuvant therapy data, (8) follow-up data. Recurrence-free survival (RFS) was defined as the interval between surgery and the first recurrence or the end of the follow-up period.

### Construction, validation, and application of the nomogram

Continuous variables were converted into categorical variables based on optimal cutoff values. The least absolute shrinkage and selection operator (LASSO) logistic regression, performed over 1000 bootstrap iterations, was used to identify variables associated with recurrence while avoiding multicollinearity in the training cohort [13]. Variables identified through LASSO logistic regression were subsequently included in the multivariate Cox proportional hazards analysis to identify independent risk factors for recurrence, which were then used to construct the nomogram. To validate the nomogram’s accuracy, the area under the receiver operating characteristic (ROC) curve (AUC) was calculated for both the training and validation cohorts, assessing its ability to predict recurrence at different time points. The nomogram’s predictive efficacy was compared to Lin’s nomogram [9]. Calibration curves were constructed to evaluate the difference between the nomogram-predicted recurrence probabilities and observed outcomes at different time points in both the training and validation cohorts. Decision curve analysis was performed to determine the net clinical benefit of the nomogram across a range of threshold probabilities in the training and validation cohorts. Using the nomogram, high-risk patients were further classified into two groups: very high-risk and general high risk. The implications of this classification for adjuvant treatment were then analyzed.

### Statistical analysis

Statistical analyses were conducted using R software version 4.4.1. Continuous variables were expressed as mean ± standard deviation or median (interquartile range) and analyzed using the Student’s *t* test or Mann–Whitney *U* test, depending on the data distribution. Categorical variables were expressed as frequencies (percentages) and compared using Pearson’s χ^2^ test. Kaplan–Meier (KM) survival curves were generated, and log-rank tests were performed to analyze the differences in RFS between groups. A *P* value <0.05 was considered statistically significant.

## Results

### Baseline characteristics and outcomes

**Figure 1.**
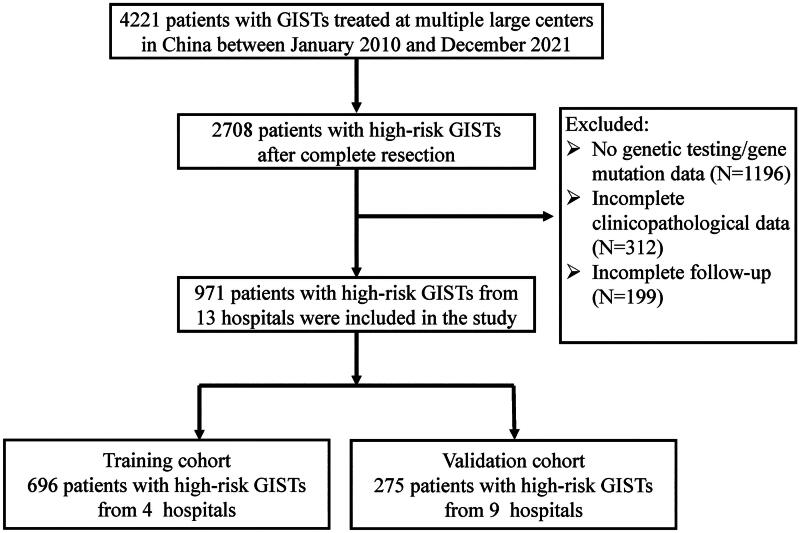
Patient selection and distribution flowchart.

Based on the inclusion and exclusion criteria, a total of 971 patients with high-risk GIST who underwent genetic testing after complete resection were enrolled in this study. Among them, 696 patients were assigned to the training cohort and 275 to the validation cohort (Figure 1). The lymphocyte count, TP, albumin (ALB), and PNI were significantly higher in the training cohort than those in the validation cohort, whereas the PLR, PAR, fibrinogen (FIB), transfusion rate, and tumor rupture rate were significantly lower. Additionally, significant differences were observed in gene mutation distributions and the duration of adjuvant therapy. However, no significant differences were observed in other clinicopathological indicators (Table 1). The median follow-up period for the study population was 49 months (33–71 months). The 3-year and 5-year RFS rates were 90.8% and 85.1%, respectively, in the training cohort and 84.1% and 75.7%, respectively, in the validation cohort.

**Table 1.** Baseline characteristics and clinicopathologic variables.

Characteristics	Total (*n* = 971)	Training cohort (*n* = 696)	Validation cohort (*n* = 275)	*F/t*	*P*
Age (years)	56.98 ± 11.72	57.28 ± 12.02	56.22 ± 10.90	1.272	0.204
Sex				1.857	0.173
Male	542 (55.8%)	398 (57.2%)	144 (52.4%)		
Female	429 (44.2%)	298 (42.8%)	131 (47.6%)		
ACCI				3.165	0.205
0–1	437 (45%)	301 (43.2%)	136 (49.5%)		
2–3	420 (43.3%)	312 (44.8%)	108 (39.3%)		
>3	114 (11.7%)	83 (11.9%)	31 (11.3%)		
WBC (10^9^/L)	6.65 ± 2.60	6.71 ± 2.51	6.49 ± 2.82	1.137	0.256
RBC (10^12^/L)	3.93 ± 0.78	3.95 ± 0.76	3.87 ± 0.83	1.348	0.178
PLT (10^9^/L)	238.09 ± 95.72	235.91 ± 95.90	243.61 ± 96.23	1.131	0.258
Hb (g/L)	113.21 ± 36.49	114.63 ± 39.39	109.61 ± 27.56	1.931	0.054
NEU (10^9^/L)	4.53 ± 2.59	4.54 ± 2.54	4.51 ± 2.72	0.171	0.864
MON (10^9^/L)	0.51 ± 0.26	0.50 ± 0.23	0.51 ± 0.32	0.562	0.574
LYM (10^9^/L)	1.68 ± 0.83	1.80 ± 0.88	1.37 ± 0.61	8.785	<0.001
PLR	190.17 ± 252.23	178.95 ± 277.28	218.58 ± 170.55	2.210	0.027
NLR	3.88 ± 7.06	3.64 ± 7.19	4.50 ± 6.69	1.705	0.088
PAR	5.06 ± 6.37	4.68 ± 6.82	6.03 ± 4.93	2.998	0.003
ALT (U/L)	20.78 ± 24.76	21.34 ± 27.40	19.36 ± 16.26	1.126	0.260
AST (U/L)	21.72 ± 28.53	22.27 ± 32.76	20.32 ± 12.45	0.962	0.336
TBI L(umol/L)	12.02 ± 10.18	11.84 ± 9.29	12.50 ± 12.15	0.914	0.361
DBIL (umol/L)	5.10 ± 7.41	5.07 ± 5.86	5.17 ± 10.36	0.184	0.854
TP (g/L)	64.97 ± 9.10	66.43 ± 8.20	61.28 ± 10.19	7.481	<0.001
ALB (g/L)	40.05 ± 6.50	40.90 ± 6.42	37.92 ± 6.21	6.565	<0.001
PNI	48.45 ± 8.79	49.91 ± 8.92	44.76 ± 7.27	9.301	<0.001
FIB (g/L)	3.80 ± 2.05	3.27 ± 1.26	5.15 ± 2.87	10.49	<0.001
Transfusion				17.744	<0.001
No	693 (71.4%)	470 (67.5%)	223 (81.1%)		
Yes	278 (28.6%)	226 (32.5%)	52 (18.9%)		
Tumor location				1.150	0.563
Stomach	426 (43.9%)	300 (43.1%)	126 (45.8%)		
Small intestine	352 (36.3%)	252 (36.2%)	100 (36.4%)		
other	193 (19.9%)	144 (20.7%)	49 (17.8%)		
Tumor size(cm)				0.756	0.385
≤8.4	540 (55.6%)	381 (54.7%)	159 (57.8%)		
>8.4	431 (44.4%)	315 (45.3%)	116 (42.2%)		
Mitotic count (/50HPF)				0.307	0.579
≤7	523 (53.9%)	371 (53.3%)	152 (55.3%)		
>7	448 (46.1%)	325 (46.7%)	123 (44.7%)		
Tumor rupture				14.038	<0.001
No	928 (95.6%)	676 (97.1%)	252 (91.6%)		
Yes	43 (4.4%)	20 (2.9%)	23 (8.4%)		
Ki-67 (%)				2.587	0.108
≤17	743 (76.5%)	523 (75.1%)	220 (80.0%)		
>17	228 (23.5%)	173 (24.9%)	55 (20.0%)		
Gene mutation				12.048	0.034
KIT exon 9	110 (11.3%)	74 (10.6%)	36 (13.1%)		
KIT exon 11 sub/dup/ Ins/del. Inv. 1 codon ex. 557/8/KIT exon 13/KIT exon 17	348 (35.8%)	239 (34.3%)	109 (39.6%)		
KIT exon 11 del. inv. 557/8	308 (31.7%)	238 (34.2%)	70 (25.5%)		
KIT exon 11 del. Inv. ≥2 codons ex. 557/8	124 (12.8%)	94 (13.5%)	30 (10.9%)		
PDGFRA	34 (3.5%)	23 (3.3%)	11 (4.0%)		
wild-type	47 (4.8%)	28 (4.0%)	19 (6.9%)		
Adjuvant therapy duration (years)				109.562	<0.001
0	223 (23.0%)	197 (28.3%)	26 (9.5%)		
0 < x ≤ 2.5	302 (31.1%)	152 (21.8%)	150 (54.5%)		
2.5 < x ≤ 3.5	163 (16.8%)	128 (18.4%)	35 (12.7%)		
3.5 < x ≤ 5	174 (17.9%)	141 (20.3%)	33(12.0%)		
x > 5	109 (11.2%)	78 (11.2%)	31 (11.3%)		

### Factors selection and nomogram development

**Figure 2.**
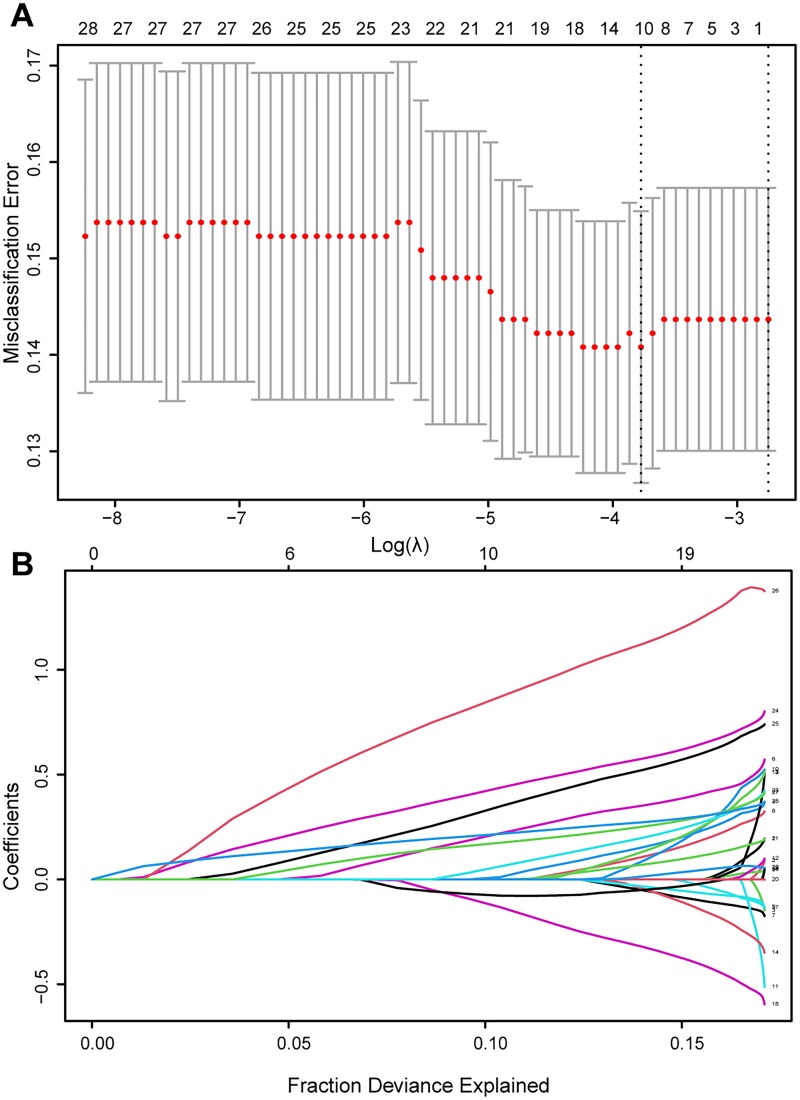
Predictors selection with LASSO logistic regression. (A) Tuning parameter selection in the LASSO logistic regression. (B) The coefficient profile plot.

In the training cohort, LASSO logistic regression incorporated 28 clinicopathological factors, of which 10 variables were selected after 1000 bootstrap iterations. The identified variables included WBC, PLT, TP, ALB, tumor location, tumor size, mitotic count, tumor rupture, Ki-67, and gene mutations. The minimum lambda value was determined to be 0.023 (Figure 2). The best cutoff values for continuous variables were as follows: WBC, PLT, TP, ALB, tumor size, mitotic count, and Ki-67 were 6.18 × 10^9^/L, 229.5 × 10^9^/L, 61.3 g/L, 36.1 g/L, 8.4 cm, 7.5/50HPF, and 17.5%, respectively.

**Figure 3.**
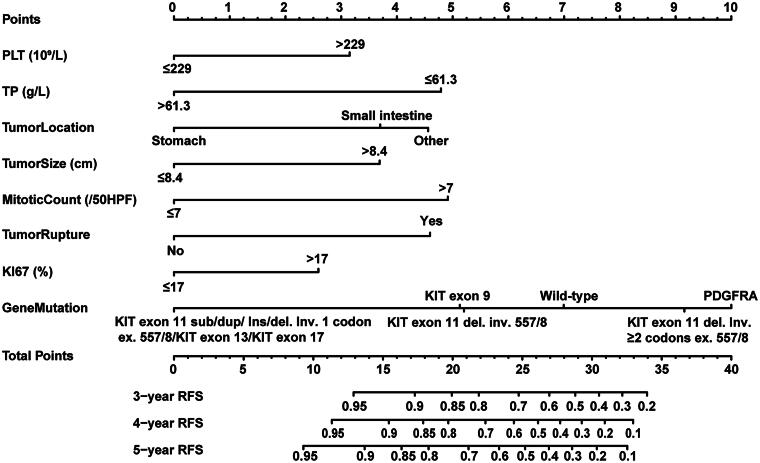
The nomogram for predicting recurrence in patients with high-risk GISTs.

These 10 variables, along with the duration of adjuvant therapy, were subsequently included in a multivariate Cox proportional hazards analysis. The results identified PLT, TP, tumor location, tumor size, mitotic count, tumor rupture, Ki-67, gene mutation, and duration of adjuvant therapy as independent prognostic predictors for patients with high-risk GIST following complete resection. All prognostic variables, except for the duration of adjuvant therapy, were used to construct the nomogram (Table 2). The developed nomogram is illustrated in Figure 3.

**Table 2.** Multivariate COX regression analysis of RFS in training dataset.

Characteristics	β coefficient	Hazard ratio (95%CI)		*P* value	Score
PLT (10⁹/L)					
≤229	reference		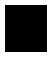		0
>229	0.659	1.932 (1.249–2.990)	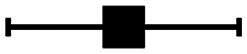	0.003	3.2
TP (g/L)					
≤61.3	reference		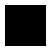		4.8
>61.3	−0.716	0.489 (0.319–0.749)	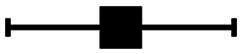	0.001	0
Tumor location					
Stomach	reference		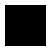		0
Small intestine	0.826	2.285 (1.360–3.838)	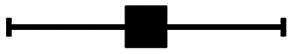	0.002	3.7
Other	0.910	2.484 (1.409–4.379)	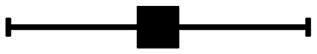	0.002	4.6
Tumor size (cm)					
≤8.4	reference		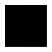		0
>8.4	0.739	2.093 (1.362–3.217)	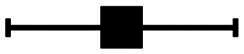	<0.001	3.7
Mitotic count (/50HPF)					
≤7	reference		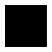		0
>7	0.865	2.376 (1.470–3.840)	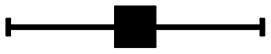	<0.001	4.9
Tumor rupture					
No	reference		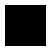		0
Yes	0.978	2.660 (1.209–5.852)	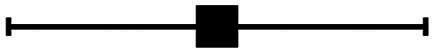	0.015	4.6
Ki-67(%)					
≤17	reference	sub/dup/Ins/del.	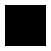		0
>17	0.478	1.612 (1.025–2.537)	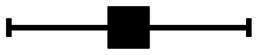	0.039	2.6
Gene mutation					
KIT exon 11 Inv. 1 codon ex. 557/8/KIT exon 13/KIT exon 17	reference		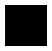		0
KIT exon 9	1.030	2.800 (1.193–6.572)	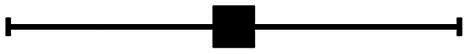	0.018	5.1
KIT exon 11 del. inv. 557/8	1.274	3.574 (1.826–6.998)	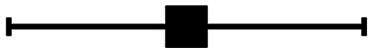	<0.001	5.2
KIT exon 11 del. Inv.≥2 codons ex. 557/8	1.476	4.374 (2.167–8.830)	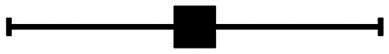	<0.001	9.2
PDGFRA	1.731	5.644 (1.916–16.619)	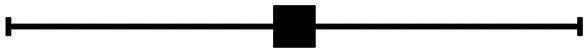	0.002	10
Wild-type	1.158	3.183 (0.995–10.188)	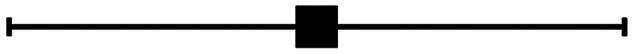	0.051	7
			0.2 0.5 1 2 5 10 20		

### Internal and external data validation of nomogram

**Figure 4.**
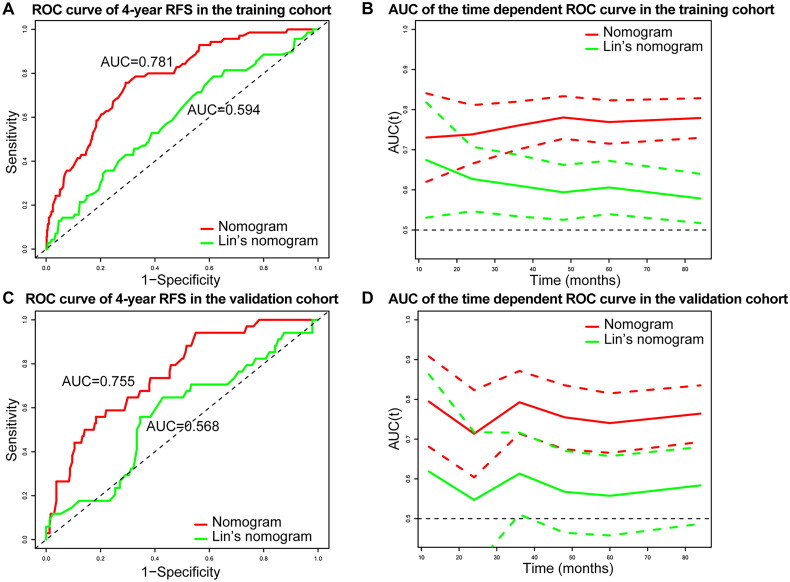
ROC curves and time-dependent ROC curves with 95% CI for the nomogram vs. Lin’s nomogram in the training and validation cohorts. (A) ROC curve of 5-year RFS in the training cohort. (B) AUC of the time dependent ROC curve in the training cohort. (C) ROC curve of 5-year RFS in the validation cohort. (D) AUC of the time dependent ROC curve in the validation cohort.

**Figure 5.**
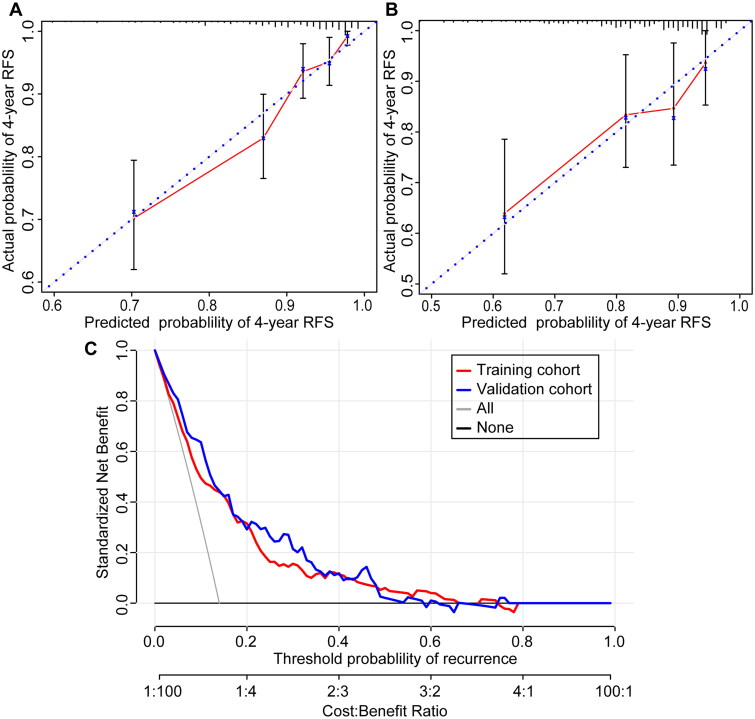
The calibration and DCA curves. (A) Calibration curve of 5-year RFS in the training cohort. (B) Calibration curve of 5-year RFS in the validation cohort. (C) DCA curve in the training and validation cohorts.

In the training cohort, the C-index of the nomogram was 0.758, and the ROC curve analysis showed that the nomogram predicted 5-year RFS with an AUC of 0.781, which was significantly higher than that of Lin’s nomogram (0.781 vs. 0.594, *p* < 0.001). Similarly, in the validation cohort, the C-index was 0.841, and the AUC was 0.755, both significantly higher than those of Lin’s nomogram (0.755 vs. 0.568, *p* < 0.001) (Figure 4). Time-dependent ROC curves confirmed that the predictive performance of the developed nomogram was significantly better than that of Lin’s nomogram in both the training and validation cohorts (Figure 4). Calibration curves demonstrated strong agreement between nomogram-predicted and observed 3-year, 4-year, and 5-year RFS rates in the training and validation cohorts (Figure 5, Supplementary Figure 1). Decision curve analysis (DCA) further validated the clinical utility of the nomogram. The DCA curve indicated that when the threshold probability of recurrence was approximately 0–60%, the nomogram provided a significant clinical benefit in guiding treatment decisions compared to treating all patients or withholding treatment entirely (Figure 5).

### Recurrence risk stratification by nomogram and its clinical guidance

**Figure 6.**
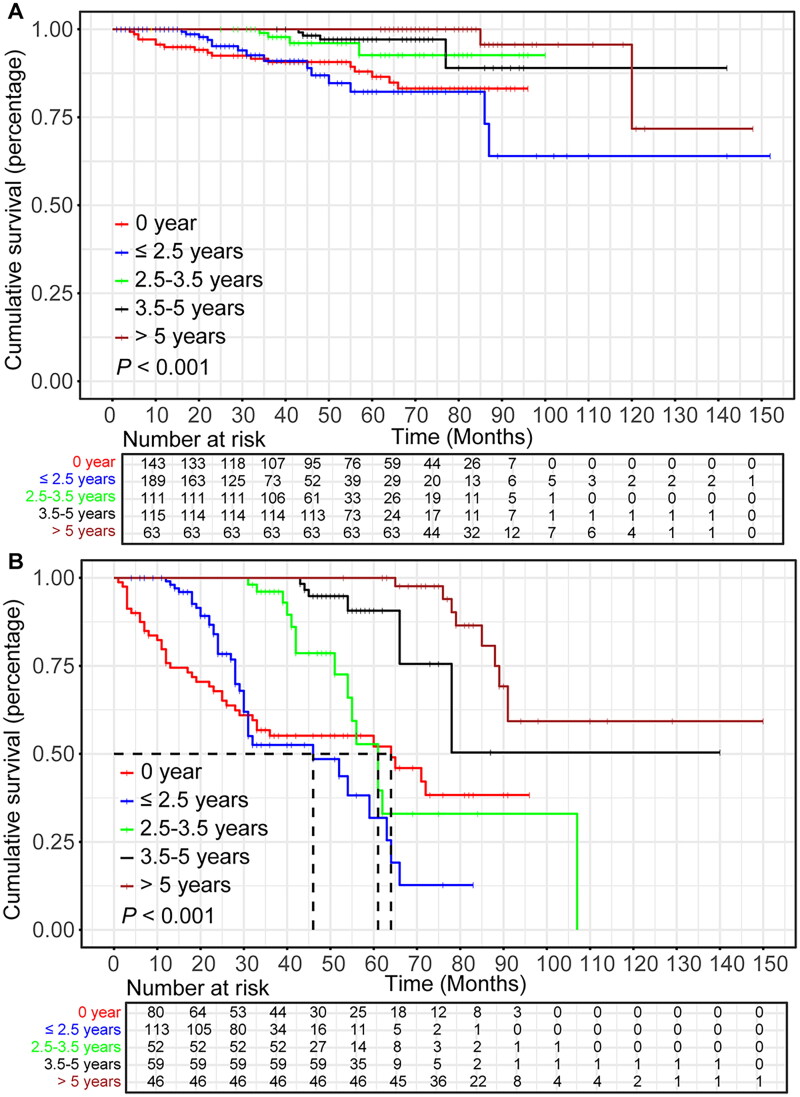
The KM curves of RFS. (A) The KM curves of RFS in the general high-risk group. (B) The KM curves of RFS in the very high-risk group.

Patients with high-risk GIST were categorized into two groups based on the optimal cut-off value of the nomogram score: those with a score ≤16.6 were in the general high-risk group, and those with a score >16.6 were in the very high-risk group. KM survival analysis demonstrated a significant difference in RFS between the two risk groups in both the training and validation cohorts (*p* < 0.001) (Supplementary Figure 2). To further investigate the effect of adjuvant therapy duration on prognosis, subgroup analyses were conducted separately within the general high-risk and very high-risk groups. The KM curves showed that in the general high-risk group, patients who received ≥2.5 years of adjuvant imatinib had a significantly better RFS than those who received <2.5 years or no adjuvant imatinib (*p* < 0.001). However, no significant difference in RFS was observed among patients who received 2.5–3.5 years, 3.5–5 years, or more than five years of adjuvant imatinib. (*p* > 0.05). In the very high-risk group, RFS was significantly better in patients with ≥ 2.5 years of adjuvant imatinib than those who received <2.5 years or no adjuvant imatinib (*p* < 0.001), and was significantly improved in patients with 3.5–5 years of adjuvant imatinib than in those with 2.5–3.5 years (*p* < 0.001). However, patients who received more than 5 years of adjuvant imatinib had the best RFS, significantly outperforming all other groups (*p* < 0.001) (Figure 6).

## Discussion

Accurately determining the probability of recurrence in patients with high-risk GIST and identifying very high-risk GIST patients with significantly poorer prognoses is crucial for optimizing treatment strategies and improving RFS. In this study, we developed and validated a multiparameter recurrence prediction nomogram to enable precise risk stratification and individualized treatment planning for patients with high-risk GIST. To the best of our knowledge, this is the first nomogram designed to predict the probability of recurrence of high-risk GIST based on gene mutations and the first study to provide adjuvant therapy guidance for high-risk GIST using a nomogram-based approach.

The predictive variables included in the nomogram were PLT, TP, tumor location, tumor size, mitotic count, tumor rupture, Ki-67, and gene mutations. Platelets and platelet-associated inflammatory markers have been reported to be significantly associated with patient prognosis in various malignancies, such as gastric cancer [14,15]. TP primarily reflects the nutritional status of patients. Ding et al. demonstrated that GIST patients with poor nutritional status had a significantly poorer prognosis [16]. In addition to TP, other nutritional markers such as sarcopenia, hemoglobin, and fibrinogen–albumin ratio have also been identified as key prognostic indicators [8,17,18]. Tumor location and size, mitotic count, and tumor rupture are generally recognized as prognostic predictors in patients with GIST and have been incorporated into the modified NIH classification system [2–10]. Ki-67 is an important index used for assessing cell proliferative activity. Studies have shown that patients with Ki-67 ≤ 5% have a significantly better prognosis than those with Ki-67 > 5% across low-risk, intermediate-risk, and overall GIST populations [3,19,20].

Genetic testing plays a critical role in GIST diagnosis, guiding targeted therapy, and evaluating treatment efficacy and prognosis. Joensuu et al. reported that patients with GIST after complete resection with *KIT* 11 deletion, indel, substitution, duplication, or insertion mutations were more responsive to imatinib and had relatively better OS, whereas patients with *KIT* 9 mutations exhibited relative imatinib resistance and poor prognosis [21]. Similarly, Shen et al. demonstrated that high-risk GIST patients with *KIT* exon 11 homozygous mutations or intron 10/exon 11 junction deletions, *KIT* exon 9 mutations, *KIT* exon 11 deletions involving codons 557/558, or deletions involving two or more codons (excluding codons 557/558) had significantly worse outcomes compared to other subgroups [12]. Our findings align with previous studies, further confirming the prognostic impact of specific genetic alterations. In addition, patients with *PDGFRA* mutations and wild-type GIST exhibited a relatively poor prognosis in this study.

A nomogram is a practical tool that integrates clinical variables to provide a simple and rapid predictive model based on complex statistical analyses. Gold et al. were the first to construct a nomogram for predicting RFS in patients with primary GIST after complete resection, utilizing pathological data in 2009 [7]. Subsequently, Lin et al. developed a nomogram for predicting RFS in patients with high-risk GIST by integrating pathological data with preoperative hematology parameters, demonstrating superior predictive performance compared to Gold’s nomogram [9]. In this study, we included gene mutations into the nomogram construction, and the results showed that the AUC values in both the training and validation cohorts were significantly higher than those in Lin’s nomogram. Calibration and DCA curves confirmed favorable discrimination, calibration accuracy, and clinical benefits. Moreover, the classification into general high-risk and very high-risk groups revealed significantly different prognoses, further validating the nomogram’s efficacy in accurately stratifying high-risk patients.

The optimal duration of adjuvant imatinib for high-risk GIST has been a growing focus of research in recent years. The PERSIST-5 study reported 5-year RFS and OS rates of 90% and 95%, respectively, in patients with high-risk GIST who received 5-year adjuvant imatinib [22]. Kang et al. revealed that primary GIST patients with tumor rupture who underwent 5-year adjuvant imatinib had significantly better RFS than those who underwent 3-year adjuvant therapy [23]. Nishida et al. found extended adjuvant therapy significantly improves RFS compared to 3 years of adjuvant therapy in patients with high-risk GIST [24]. Bertsimas et al. developed an optimal policy tree for patients with intermediate- and high-risk GIST and indicated that 5 years is the optimal duration of imatinib treatment [25]. The IMADGIST study compared the efficacy of 6 years and 3 years of adjuvant imatinib in patients with high risk GIST and found that three additional years of adjuvant imatinib reduces the risk of relapse with an acceptable tolerance [26]. In this study, 3 years of adjuvant imatinib was found to be sufficient for patients with general high-risk GIST, while 5 years or more of adjuvant imatinib is recommended for patients with very high-risk GIST. These findings provide valuable guidance for individualized treatment strategies in patients with high-risk GIST and help prevent unnecessary prolongation of adjuvant therapy, which could lead to increased drug toxicity and economic burden.

This study had several limitations. As with all retrospective studies, the current investigation is inherently subject to selection bias, which must be acknowledged as a methodological limitation. Furthermore, our study cohort was exclusively drawn from mainland Chinese populations, which may restrict the generalizability of our findings to other ethnic groups or healthcare settings. These limitations underscore the need for validation through international multicenter prospective studies to confirm our conclusions. In addition, some gene mutations, such as *KIT* exon 13 or 17 mutations, were identified in a small number of patients, making it difficult to analyze in separate groups.

## Conclusion

As the largest nomogram-related multicenter study in high-risk GIST, this study developed and validated a novel nomogram for accurately predicting recurrence based on clinicopathological indicators and gene mutations and provided guidance on adjuvant therapy for the first time. For patients with general high-risk GIST, 3 years of adjuvant imatinib is adequate, whereas patients with very high-risk GIST need more than 5 years of adjuvant imatinib. These findings are essential for improving the efficacy of adjuvant therapy, enhancing the prognosis of patients with high-risk GIST, and preventing unnecessary medication exposure.

## Ethics approval

The study was approved by the Ethics Committee of Union Hospital, Tongji Medical College, Huazhong University of Science and Technology (approval number: 20230523), which confirmed that informed consent should be waived because this study was a retrospective study and did not compromise patient privacy or cause harm to patients, and was conducted in compliance with the Declaration of Helsinki.

## Supplementary Material

Supplemental Material

Supplementary FIG 2.tif

Supplementary FIG 1.tif

## Data Availability

The data analysed in this study can be made available by the corresponding author on reasonable request.

## Abbreviations

GIST: Gastrointestinal stromal tumor
LASSO: Least absolute shrinkage and selection operator
PLT: Platelet
TP: Total protein
RFS: Recurrence-free survival
ACCI: Age-adjusted Charlson comorbidity index
WBC: White blood cell count
RBC: Red blood cell count
PLR: Platelet–lymphocyte ratio
NLR: Neutrophil–lymphocyte ratio
PAR: Platelet–albumin ratio
PNI: Prognostic nutritional index
ROC: Receiver operating characteristic
AUC: Area under the receiver operating characteristic curve
KM: Kaplan–Meier
ALB: Albumin
FIB: Fibrinogen
DCA: Decision curve analysis

## References

[1] Dermawan JK, Rubin BP. Molecular pathogenesis of gastrointestinal stromal tumor: a paradigm for personalized medicine. Annu Rev Pathol. 2022;17(1):323–344. doi: 10.1146/annurev-pathol-042220-021510. 34736340

[2] Keun Park C, Lee EJ, Kim M, et al. Prognostic stratification of high-risk gastrointestinal stromal tumors in the era of targeted therapy. Ann Surg. 2008;247(6):1011–1018. doi: 10.1097/SLA.0b013e3181724f9d. 18520229

[3] Liu X, Qiu H, Wu Z, China Gastrointestinal Stromal Tumor Study Group (CN-GIST)., et al. A novel pathological prognostic score (pps) to identify “very high-risk” patients: a multicenter retrospective analysis of 506 patients with high risk gastrointestinal stromal tumor (GIST). J Gastrointest Surg. 2018;22(12):2150–2157. doi: 10.1007/s11605-018-3799-5. 30030719

[4] Zheng J, Li R, Qiu H, et al. Tumor necrosis and >20 mitoses per 50 high-power fields can distinguish ‘very high-risk’ and ‘highest-risk’ within ‘high-risk’ gastric gastrointestinal stromal tumor. Fut Oncol. 2018;14(7):621–629. doi: 10.2217/fon-2017-0509. 29411689

[5] Xu SJ, Zhang SY, Dong LY, et al. Dynamic survival analysis of gastrointestinal stromal tumors (GISTs): a 10-year follow-up based on conditional survival. BMC Cancer. 2021;21(1):1170. doi: 10.1186/s12885-021-08828-y. 34724907 PMC8559392

[6] Wang L, Ni Z, Xu W, et al. Clinical characteristics and outcomes of gastrointestinal stromal tumor patients receiving surgery with or without TKI therapy: a retrospective real-world study. World J Surg Oncol. 2023;21(1):21. doi: 10.1186/s12957-023-02897-y. 36691015 PMC9869533

[7] Gold JS, Gönen M, Gutiérrez A, et al. Development and validation of a prognostic nomogram for recurrence-free survival after complete surgical resection of localised primary gastrointestinal stromal tumour: a retrospective analysis. Lancet Oncol. 2009;10(11):1045–1052. doi: 10.1016/S1470-2045(09)70242-6. 19793678 PMC3175638

[8] Lee CK, Goldstein D, Gibbs E, et al. Development and validation of prognostic nomograms for metastatic gastrointestinal stromal tumour treated with imatinib. Eur J Cancer. 2015;51(7):852–860. doi: 10.1016/j.ejca.2015.02.015. 25801699

[9] Lin Y, Wang M, Jia J, et al. Development and validation of a prognostic nomogram to predict recurrence in high-risk gastrointestinal stromal tumour: a retrospective analysis of two independent cohorts. EBioMed. 2020;60:103016. doi: 10.1016/j.ebiom.2020.103016. PMC7522759 32980695

[10] Bertsimas D, Margonis GA, Tang S, et al. An interpretable AI model for recurrence prediction after surgery in gastrointestinal stromal tumour: an observational cohort study. E Clin Med. 2023;64:102200. doi: 10.1016/j.eclinm.2023.102200. PMC10507206 37731933

[11] Koppie TM, Serio AM, Vickers AJ, et al. Age-adjusted Charlson comorbidity score is associated with treatment decisions and clinical outcomes for patients undergoing radical cystectomy for bladder cancer. Cancer. 2008;112(11):2384–2392. doi: 10.1002/cncr.23462. 18404699

[12] Shen YY, Ma XL, Wang M, et al. Exon 11 homozygous mutations and intron 10/exon 11 junction deletions in the KIT gene are associated with poor prognosis of patients with gastrointestinal stromal tumors. Cancer Med. 2020;9(18):6485–6496. doi: 10.1002/cam4.3212. 32697050 PMC7520349

[13] Sun K, Huang SH, Wong DS, et al. Design and application of a variable selection method for multilayer perceptron neural network with LASSO. IEEE Trans Neural Netw Learn Syst. 2017;28(6):1386–1396. doi: 10.1109/TNNLS.2016.2542866. 28113826

[14] Shi H, Wang H, Pan J, et al. Comparing prognostic value of preoperative platelet indexes in patients with resectable gastric cancer. Sci Rep. 2022;12(1):6480. doi: 10.1038/s41598-022-10511-6. 35444195 PMC9021185

[15] Li Z, Li S, Ying X, et al. The clinical value and usage of inflammatory and nutritional markers in survival prediction for gastric cancer patients with neoadjuvant chemotherapy and D2 lymphadenectomy. Gastric Cancer. 2020;23(3):540–549. doi: 10.1007/s10120-019-01027-6. 32072387 PMC7165147

[16] Ding P, Guo H, Sun C, et al. Relationship between nutritional status and clinical outcome in patients with gastrointestinal stromal tumor after surgical resection. Front Nutr. 2022;9:818246. doi: 10.3389/fnut.2022.818246. 35187038 PMC8847716

[17] Song H, Xiao X, Liu G, et al. Sarcopenia as a novel prognostic factor in the patients of primary localized gastrointestinal stromal tumor. BMC Cancer. 2022;22(1):179. doi: 10.1186/s12885-022-09278-w. 35177018 PMC8851766

[18] Li R, Song S, He X, et al. Relationship between fibrinogen to albumin ratio and prognosis of gastrointestinal stromal tumors: a retrospective cohort study. Cancer Manag Res. 2020;12:8643–8651. doi: 10.2147/CMAR.S271171. 32982455 PMC7509338

[19] Cao L, Lin C, Liu Y, et al. Clinical characteristics and prognostic analysis of postoperative recurrence or metastasis of low-risk gastrointestinal stromal tumors. World J Surg Oncol. 2024;22(1):65. doi: 10.1186/s12957-024-03339-z. 38395931 PMC10885449

[20] Hu X, Zhang Q, Wang Z, et al. Retrospective study of the clinicopathological characteristics and prognostic factors of gastrointestinal stromal tumors in Chinese patients. Ann Diagn Pathol. 2022;61:152050. doi: 10.1016/j.anndiagpath.2022.152050. 36257237

[21] Joensuu H, Wardelmann E, Eriksson M, et al. KIT and PDGFRA mutations and survival of gastrointestinal stromal tumor patients treated with adjuvant imatinib in a randomized trial. Clin Cancer Res. 2023;29(17):3313–3319. doi: 10.1158/1078-0432.CCR-22-3980. 37014660 PMC10472091

[22] Raut CP, Espat NJ, Maki RG, et al. Efficacy and tolerability of 5-year adjuvant imatinib treatment for patients with resected intermediate- or high-risk primary gastrointestinal stromal tumor: the PERSIST-5 Clinical Trial. JAMA Oncol. 2018;4(12):e184060. doi: 10.1001/jamaoncol.2018.4060. 30383140 PMC6440723

[23] Kang S, Ryu MH, Bang YH, et al. 5 years versus 3 years in patients with ruptured localized gastrointestinal stromal tumor: a retrospective analysis. Cancer Res Treat. 2022;54(4):1167–1174. doi: 10.4143/crt.2021.1040. 34883555 PMC9582464

[24] Nishida T, Sato S, Ozaka M, et al. Long-term adjuvant therapy for high-risk gastrointestinal stromal tumors in the real world. Gastric Cancer. 2022;25(5):956–965. doi: 10.1007/s10120-022-01310-z. 35672526

[25] Bertsimas D, Margonis GA, Sujichantararat S, et al. Interpretable artificial intelligence to optimise use of imatinib after resection in patients with localised gastrointestinal stromal tumours: an observational cohort study. Lancet Oncol. 2024;25(8):1025–1037. doi: 10.1016/S1470-2045(24)00259-6. 38976997 PMC12051465

[26] Blay JY, Schiffler C, Bouché O, et al. A randomized study of 6 versus 3 years of adjuvant imatinib in patients with localized GIST at high risk of relapse. Ann Oncol. 2024;35(12):1157–1168. doi: 10.1016/j.annonc.2024.08.2343. 39241959

