# Supplementary material for: Distinguishing very high-risk patients among high-risk gastrointestinal stromal tumor cases: development and validation of a nomogram based on a multicenter population-based retrospective cohort study
Source: Ann Med. 2025 Jun 20;57(1):2520896. doi: 10.1080/07853890.2025.2520896 (PMC12931352; doi:10.1080/07853890.2025.2520896)
Supplement: Supplemental Material [file IANN_A_2520896_SM0885.docx]

***Supplementary Figure 1***. The calibration curves. A. The calibration curves of 3-year RFS in the training cohort. B. The calibration curves of 3-year RFS in the validation cohort. C. The calibration curves of 7-year RFS in the training cohort. D. The calibration curves of 7-year RFS in the validation cohort.

***Supplementary Figure 2.*** The KM curves of RFS. A. The KM curves of RFS for patients with general high-risk and very high-risk GIST in the training cohort. B. The KM curves of RFS for patients with general high-risk and very high-risk GIST in the validation cohort
