# Supplementary figures and images for: Distinguishing very high-risk patients among high-risk gastrointestinal stromal tumor cases: development and validation of a nomogram based on a multicenter population-based retrospective cohort study
Source: Ann Med. 2025 Jun 20;57(1):2520896. doi: 10.1080/07853890.2025.2520896 (PMC12931352; doi:10.1080/07853890.2025.2520896)

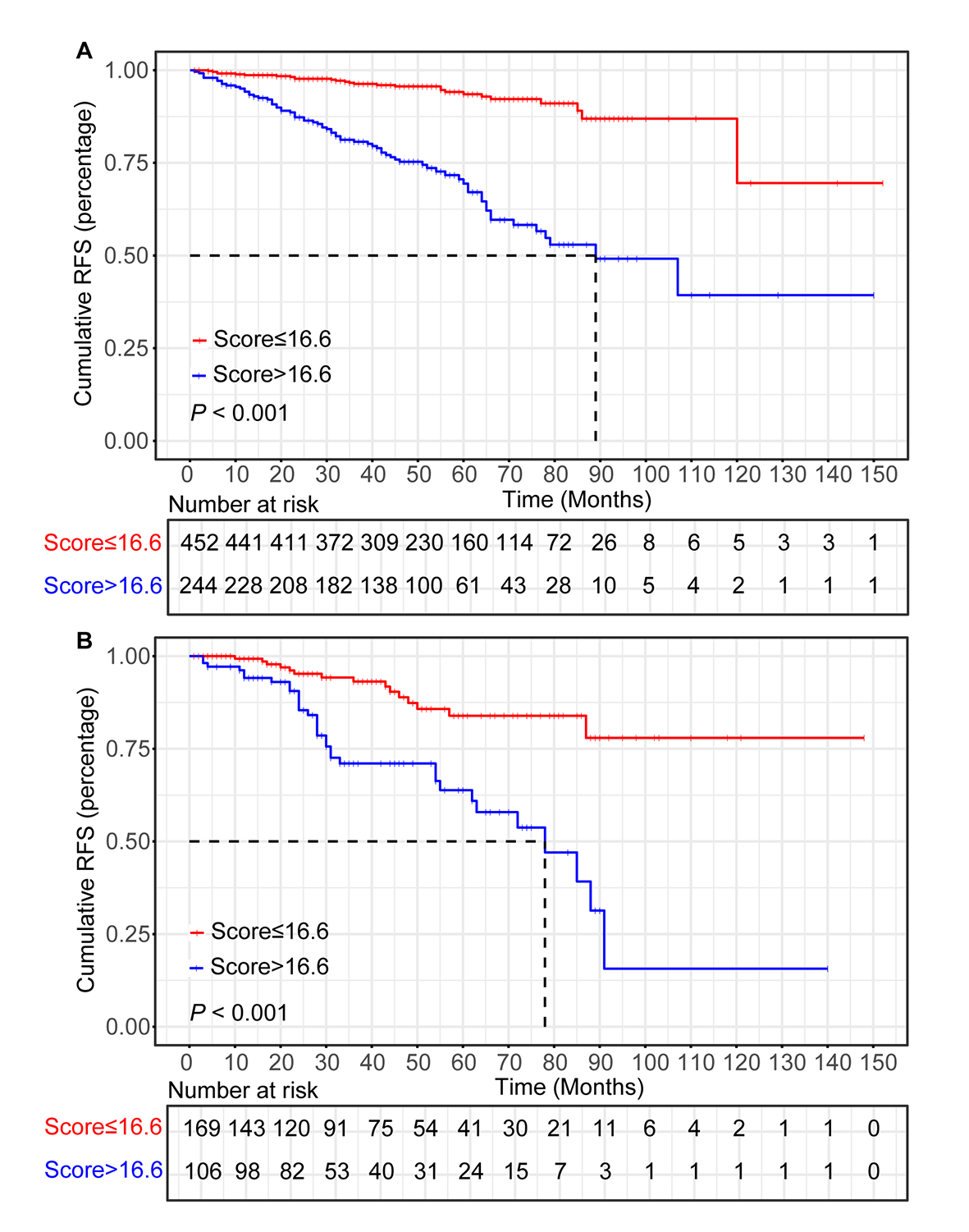

Supplement: Supplementary FIG 2.tif [file IANN_A_2520896_SM9289.tif]

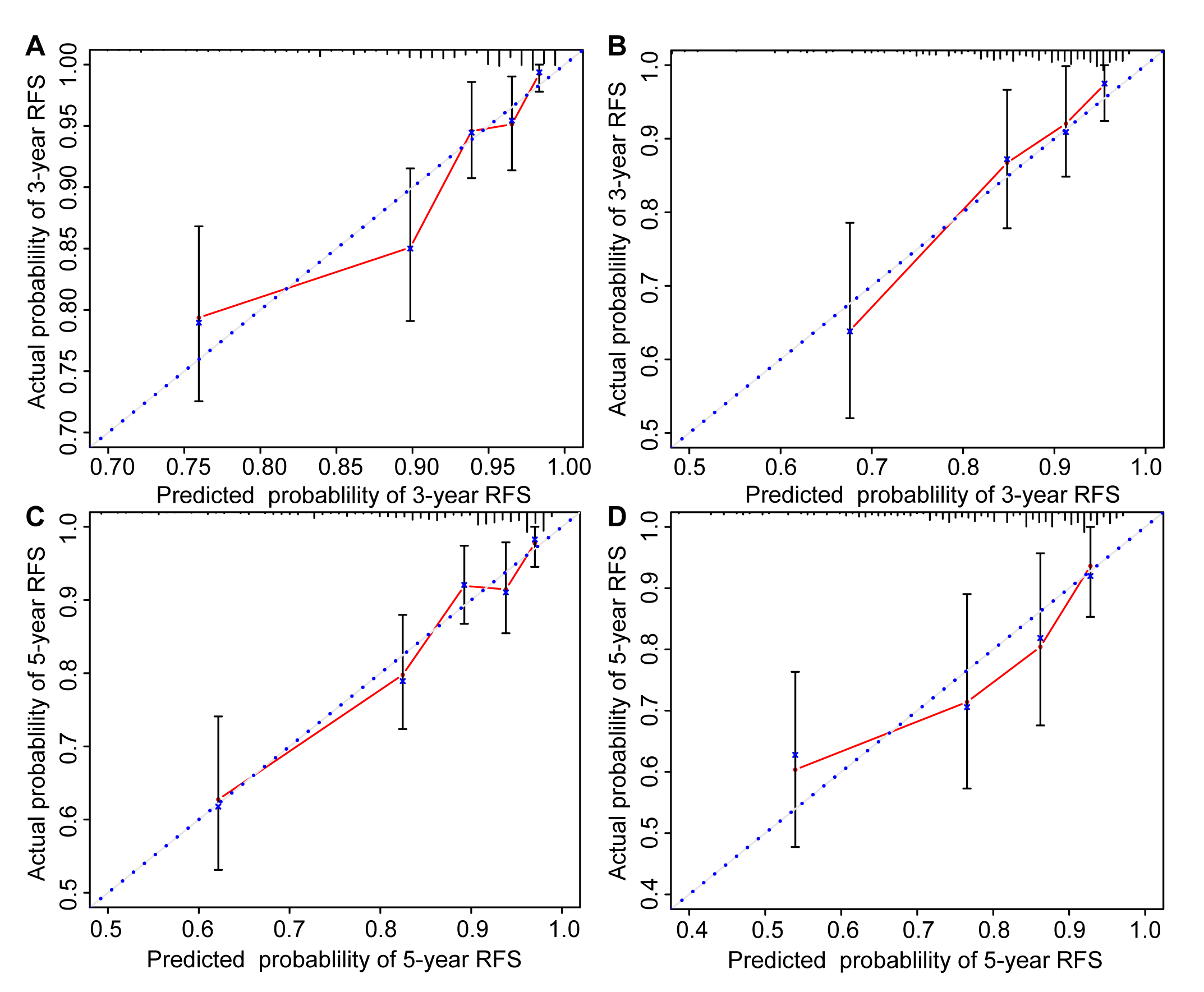

Supplement: Supplementary FIG 1.tif [file IANN_A_2520896_SM9288.tif]
